# Topical Herbal Application in the Management of Atopic Dermatitis: A Review of Animal Studies

**DOI:** 10.1155/2014/752103

**Published:** 2014-06-15

**Authors:** Younghee Yun, Kyuseok Kim, Inhwa Choi, Seong-Gyu Ko

**Affiliations:** ^1^Department of Dermatology of Korean Medicine, College of Korean Medicine, Graduate School of Kyung Hee University, Seoul 130-701, Republic of Korea; ^2^Department of Dermatology of Korean Medicine, Kyung Hee University Hospital at Gangdong, No. 149 Sangil-dong, Gangdong-gu, Seoul 134-090, Republic of Korea; ^3^Department of Ophthalmology & Otorhinolaryngology & Dermatology of Korean Medicine, College of Korean Medicine, Kyung Hee University, Seoul 130-701, Republic of Korea; ^4^Department of Preventive Medicine, College of Korean Medicine, Kyung Hee University, Seoul 130-701, Republic of Korea

## Abstract

Herbs are widely used in the treatment of atopic dermatitis (AD) in Eastern Asian countries, and certain herbs regarded have anti-inflammatory properties that can help with AD. With the goal of developing a topical herbal agent for AD, we conducted a systematic review of *in vivo* studies of AD-like skin models for screening potential herbs. Searches were conducted from PubMed and EMBASE. After all, 22 studies were included for this review. We judged most of the domains of all studies to be at unclear risk of bias. Among 22 included studies, 21 herbs have been reported to reduce AD-like skin lesions in mouse models by suppressing Th2 cell response. Our findings may offer potential herbs for the topical application treatment of AD.

## 1. Introduction

There are many chemical substances that have been derived from plants for use as drugs, and these include some of the most utilized drugs such as aspirin, atropine, digoxin, ephedrine, morphine, quinine, and taxol. The latest version of the Dictionary of Natural Products (DNP; http://dnp.chemnetbase.com/) has just over 260,000 entries. Over the past decades, natural sauces have only taken a secondary role in drug discovery and development after the advent of molecular biology and combinatorial chemistry. However, as a basis for drug development, a new interest in the role of natural sauces has been concentrated, because various “-omics” technologies now allow scientists to detail the exact biological effects of natural sauces [1].

Atopic dermatitis (AD) is a chronic inflammatory skin disease with an increasing prevalence in industrialized countries. AD is characterized by pruritus; eczematous lesions accompanied by excessive infiltration of inflammatory cells such as lymphocytes, macrophages, and granulated mast cells in the skin lesions; eosinophilia in the peripheral blood; and high levels of serum immunoglobulin IgE. Although the pathogenesis of AD has not yet been fully understood, genetic, environmental, pharmacological, psychological, immunological, and skin barrier dysfunction factors are believed to contribute to the underlying pathogenic mechanisms [2–4].

Topical steroids are commonly used to treat moderate-to-severe AD, but long-term use of steroids at high concentrations is associated with a number of side effects [5]. Among various natural sources such as plants, animals, or microorganisms, herbs are widely used in the treatment of atopic dermatitis (AD) in Eastern Asian countries, and certain herbs regarded have anti-inflammatory properties that can help with AD. Our interest is to develop a safe and curative herb derived agent for AD using medical knowledge and clinical experience of herbal medicine combining with molecular biology and combinatorial chemistry technologies.

Recently, since Sandercock and Roberts drew attention to the need for more animal studies before beginning studies in human patients [6], there has been an increasing interest in the systematic reviews of research involving animals.

Systematic reviews can aid in the development of more effective therapeutic agents for AD by extrapolating the results of animal studies to humans [7]. We performed a systematic review with this goal in mind and our objectives were (i) to screen topically applicable herbs for AD, (ii) to suggest potential mechanisms of action of topical herbal application in animal models of AD, and (iii) to ascertain the conditions of animal experiments used in the studies.

## 2. Methods

### 2.1. Criteria for Considering Studies for This Review

#### 2.1.1. Inclusion Criteria

- Studies on the use of topical herbs for AD in animal models
- Published between 2009 and 2013
- Full text available
- Article in English.

#### 2.1.2. Exclusion Criteria

- Not related to AD or allergic dermatitis
- Not an animal study
- Animal cell studies
- Not an investigational study of herbs
- Not an investigational study of herbs alone
- Studies investigating compounds isolated from herbs
- Use of fermented herbs by* Lactobacillus plantarum* and so forth
- Studies investigating oils from herbs
- Pharmacoacupuncture
- Preexisting herbal drugs
- Anal, intraperitoneal, or oral administration of herbs
- Herbal mixtures
- Biomarkers not used as outcome measurements.

### 2.2. Search Methods for Identification of Studies

#### 2.2.1. Data Sources and Searches

Literature searches were performed using PubMed and EMBASE databases. Search terms contained three components: (A) intervention/exposure, (B) disease of interest, and (C) animal species, with adjustments made for the different databases. Herbs were defined as plants, part of plants, or plant extracts that are used for medical purposes. Since the administration method of the herbs, as well as the outcome measures, was commonly described in the main article and rarely indexed in many papers, we excluded the administration method and outcome measures in the search strategy. For identification of MeSH terms, we used the PubMed thesaurus and the MeSH database, while we used EMTREE terms for searches using EMBASE. To identify all animal studies in PubMed, we used the “Animal search filter” that Hooijmans et al. [8] designed, while we used the filters in EMBASE. The full lists of search terms are presented in Tables 1 and 2.

#### 2.2.2. Selection of Studies

Two authors (Yun and Kim) independently conducted the database searches. Duplicate articles were removed by the first author (Yun). Moreover, the references lists of review articles on relevant topics were manually searched by the two authors. For identifying eligibility of each study, the two authors read all potentially relevant articles. Disagreements were resolved by discussions with the corresponding author (Choi and Ko).

## 3. Results

### 3.1. Identification of Studies

**Figure 1:**
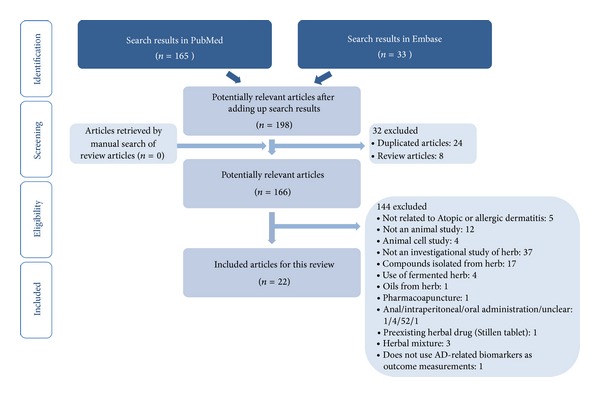
Flowchart of the study selection process.

After adding the search results from PubMed (*n* = 165) and EMBASE (*n* = 33), duplicate articles (*n* = 24) were removed. References lists in review articles (*n* = 8) were searched but did not result in any articles being retrieved. From the potentially relevant articles (*n* = 166), we excluded 144 articles based on the predefined exclusion criteria, resulting in a total of 22 studies being included in this review (Figure 1).

### 3.2. Risk of Bias

**Figure 2:**
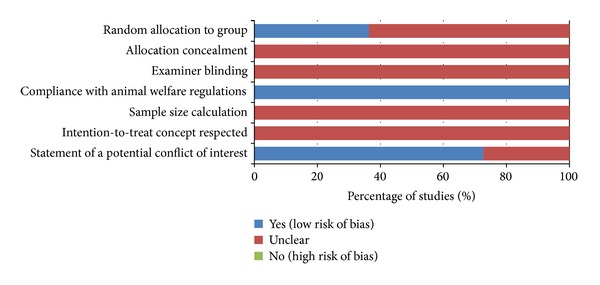
Risk of bias in the studies.

Figure 2 shows the study quality checklist items reported for each included study, including random allocation to treatment groups (*n* = 8, 36.4%), compliance with animal welfare regulations (*n* = 22, 100%), and statements of a potential conflict of interest (*n* = 16, 72.7%). None of the studies reported allocation concealment, examiner blinding, sample size calculation, and if results were based on analysis of the intent-to-treat population.

### 3.3. Basic Characteristics and Investigated Herbs in the Included Studies

Twenty-one studies were conducted in Korea and one was conducted in Japan. In two studies, herbs of the genus* Chrysanthemum* were investigated. Otherwise, there were no studies investigating the same herb (Table 4). Herbal extracts were prepared using ethanol, water, methanol, butanol, chloroform, 1,3-butylene glycol, or indirect heat.

### 3.4. Animal Models Used in the Included Studies

All studies used mice to investigate topical herbal application in an* in vivo* setting. The NC/Nga mouse (*n* = 16) was the most frequently used mouse model in these studies, followed by BALB/c (*n* = 4), C57BL/6 (*n* = 1), and hairless mice (*n* = 1).

The methods used for induction of AD-like skin lesions varied depending on the study. Repeated cutaneous application of chemical allergens and house dust mite allergens was used in 15 and 10 studies, respectively. Skin injury by stripping using surgical tape was used in 2 studies. For barrier disruption, sodium dodecyl sulfate (SDS) was applied to the lesions in 8 studies. Dorsal skin, ears, or a combination of dorsal skin and ears was used in most of the studies (Table 3).

### 3.5. Main Outcomes Investigated, Results, and Suggested Mechanisms of Action

In most of the studies, clinical symptoms, serum IgE levels, and Th1- and/or Th2-related cytokines and/or chemokines were assessed as outcome measurements (Table 4). The clinical severity of dermatitis was scored, and severity was found to have decreased after topical herbal application in 15 studies. Epidermal, dermal, or ear thickness was measured and was found to be decreased in 13 studies, indicating a decrease in the severity of the inflammatory process. Frequency of scratching was measured by counting scratching episodes in 5 studies, either directly or by reviewing videos of the animal. After topical herbal application, the frequency of scratching was decreased in all 5 studies.

Elevated serum IgE levels are important characteristics of AD. Serum or plasma IgE levels were measured in 21 studies, and, in 20 of these, serum or plasma IgE levels were decreased after the topical herbal application. However, in one study [9], neither topical* Rehmannia glutinosa* extract nor tacrolimus reduced the increased serum IgE levels after allergen sensitization, although they both suppressed the expression of interleukin- (IL-) 4 mRNA in the ear lesions. Antigen-specific IgE levels were measured in two studies, both of which used house dust mite allergen and DNCB to induce AD-like skin lesions.

Most of the included studies investigated the Th2-response suppressing effects and/or Th1-response modulating effects upon topical herbal application in the AD-like mouse models. In 14 studies, only Th2-related biomarkers were measured, while both Th1- and Th2-related biomarkers were measured in nine studies. In all 21 studies that measured Th2 responses, topical herbal application resulted in decrease of Th2-related cytokines, chemokines, proinflammatory factors, and adhesion molecules. Conversely, among the eight studies that measured interferon- (IFN-)*γ*, topical herbal application resulted in increased IFN-*γ* levels in two studies, decreased levels in five studies, and no induced changes in one study. Interestingly, in one study [10],* Chrysanthemum indicum *L. decreased both Th1 (IFN-*γ*) and Th2 cytokines (IL-4 and IL-13); however, the ratio of Th1 to Th2 cytokines was increased by herbal application.

## 4. Discussion

Herbal medicine is the use of medicinal plants for prevention and treatment of disease. Herbs and their derivatives have been, and continue to be, rich sources for drug discovery. Recently, results from several studies have indicated that patients with AD may benefit from herbal medicines [11–13]. Certain herbs are regarded to have anti-inflammatory properties that can reduce the symptoms of AD. In Asian herbal medicine, herbs are categorized according to their functions. One such group of categorized herbs is named the clear heat drug group (*凊* *熱* *藥*), and these herbs can be used for treating fever, infectious disease, and inflammatory conditions. Among the included 22 studies, seven studies [9, 10, 14–18] investigated herbs belonging to the clear heat drug group. Among these seven studies, two studies investigated herbs of the genus* Chrysanthemum*.

Since multiple genetic and environmental factors may underlie AD, the notion of developing a single comprehensive animal model is unrealistic [19]. Since the description of the Nc/Nga mouse as the first spontaneously occurring model of AD in 1997 [20], a number of mouse models have been developed. They can be classified into three groups: (1) models induced by epicutaneous application of sensitizers, (2) mice that spontaneously develop AD-like skin lesions, and (3) transgenic mice that either overexpress or lack selective molecules [21].

NC/Nga mice were used in 16 of the analyzed studies. These mice are free of dermatitis in pathogen-free conditions but develop a spontaneous AD-like eruption when conventionally housed, and they have historically been viewed as one of the best animal models for assessing this condition [19]. In other studies, models induced by epicutaneous application of sensitizers were used. However, none of the included studies used genetically engineered mouse models.

In most of the included studies, clinical symptoms, serum IgE levels, and Th1- and/or Th2-related cytokines and/or chemokines were measured as outcome measurements. Based on the decreased clinical scores, ear or epidermal thickness, scratching behaviors, and histological inflammations after herbal application, it can be hypothesized that topical herbal application has anti-inflammatory effects. However, we could not conduct a meta-analysis to integrate quantitative analyses, since the studies included in our study all investigated different types of herbs.

Elevated serum IgE levels are an important feature of AD. Several studies have demonstrated that serum IgE levels are elevated in patients with AD; furthermore, serum IgE levels have been shown to be elevated in NC/Nga mice with AD-like skin lesions [20]. Among the 22 included studies, serum or plasma IgE levels were measured in 21 studies, and, in 20 studies, serum or plasma IgE levels were decreased after herbal treatment. However, in one study [9], neither topical application of* Rehmannia glutinosa* extract nor tacrolimus reduced the increased serum IgE levels after allergen sensitization, although they both suppressed the expression of IL-4 mRNA in the ear lesions and serum. The authors discussed two possible reasons for this observation: (i) that locally expressed IL-4 in the ear lesions did not contribute to systemic IgE production or (ii) that, in a short-term study with topical herbal application, effects on serum IgE may not be observed [22]. However, we noted that the authors did not measure the levels of allergen-specific IgE, and, in general, total IgE concentrations are a relatively crude method of detecting allergic disorders, since normal values do not exclude the presence of allergic disease, particularly to a single allergen, and since elevated levels of total IgE can be found in many patients with no evidence of allergy [23].

Conversely, both total and allergen-specific IgE levels were measured and found to be decreased after topical application of water-soluble extract of* Lindera obtusiloba* and* Phellinus linteus* in two studies, which used both house dust mite allergens and DNCB to induce AD-like skin lesions [24, 25]. Furthermore, water-soluble extract of* P. linteus* did not affect the total IgG levels, and it was found to be more potent than ceramide in reducing mite-specific IgE levels. These data suggest that certain herbs can suppress allergic responses in an allergen-specific manner. Nevertheless, immunological and clinical parameters for the assessment of antigen-specific immune responses were not measured in most of the studies.

Both Th1- and Th2-type cytokines contribute to the pathogenesis of AD, and their expression patterns are not mutually exclusive [2]. Th2 cytokines such as IL-4, IL-5, and IL-13 play key roles in the hyperproduction of IgE, whereas Th1 cytokines, especially IFN-*γ*, are strong inhibitors of IgE synthesis, Th2 cell proliferation, and IL-4 receptor expression on T-cells [26]. Development of AD is induced by Th2-type responses, while the chronic inflammatory responses are dominantly mediated by Th1-type reactions.

Among the 22 included studies, 21 herbs were reported to reduce AD-like skin lesions in mouse models by suppressing Th2 cell response with or without balancing of the Th1/Th2 cell response. In eight studies, Th1 cytokines were measured and showed different results. Based on this review, it seems that investigators mainly assess Th1- and Th2-related mechanisms to explain the anti-inflammatory effects of herbs.

In the present study, out of 166 potential studies, we identified 22 studies that met all the selection criteria. It showed that there is room for methodological improvement in the studies. Most studies were at an unclear risk of bias; therefore, it was not possible to accurately determine the degree of bias of the described treatment effects. Further research should be conducted with well-designed methodological research protocols using random allocation, allocation concealment, assessor blindness, sample sizes calculation, and intention-to-treat-respected analyses.

Twenty-one studies were conducted in Korea and one was conducted in Japan. For identifying all potentially relevant researches, the search strategy in the present study included American (PubMed) and European (EMBASE) databases. No attempts were made to retrieve articles from Chinese, Korean, or Japanese databases. Also, non-English articles from PubMed and EMBASE were not included. Because of these, studies may have been excluded. However, when we made our search strategy, we did not expect different results among the countries (Korea, Japan, and China) which conduct a relatively large number of studies on herbs. We assume that the great interest in topical use of herbs and the large number of research and development (R&D) projects on herbs in Korea have been important factors of the results.

In summary, we have reviewed studies investigating topical herbal application in AD-like animal models. For all studies, we judged most domains to be at unclear risk of bias. Herbs of the genus* Chrysanthemum* were used in two studies, and seven studies investigated herbs of the clear heat drug group. Among the AD-like animal models, NC/Nga and BALB/c mice treated with chemical haptens, DNCB, DNFB, or TNCB were used in most of the studies. Clinical symptoms, serum IgE levels, and Th1- and/or Th2-related cytokines and/or chemokines were assessed as outcome measurements. Among the 22 included studies, 21 herbs were reported to reduce AD-like skin lesions in mouse models by suppressing Th2 cell responses. By summarizing the results from the published literature, we hope that this study might aid in finding a potential herbal therapeutic agent for the treatment of AD. The limitation of this study was that a meta-analysis was not conducted because of the variety of investigated herbs included in the studies. Nevertheless, this review may assist in identifying directions for further researches endeavors.

## Figures and Tables

**Table 1:** Search strategy for PubMed.

Intervention/exposure	“Herbal Medicine” [MeSH term] or “Herbals” [MeSH terms] or “Herbals as Topic” [MeSH terms] or “Plant Extracts” [MeSH terms] or “Drugs, Chinese Herbal” [MeSH term] or “Plants, Medicinal” [MeSH terms] or “Ethnobotany” [MeSH term] or “Medicine, Traditional” [MeSH Terms] or “Phytotherapy” [MeSH terms] or “Herb” [tiab] or “Extract” [tiab] or “Extracts” [tiab] or “Chinese medicine” [tiab] or “Korean medicine” [tiab] or “Kampo” [tiab]
Disease of interest	“dermatitis, atopic” [MeSH Terms] or “Atopic dermatitis” [title/abstract] or “Eczema, Atopic” [title/abstract]
Animal species	Using “Animal search filter” [8]
Outcome measures	Not included in the search strategy
Filter	Full text available AND English AND Published in the last 5 years

**Table 2:** Search strategy for EMBASE.

Intervention/exposure	Medical plant [EMTREE]
Disease of interest	Atopic dermatitis [EMTREE]
Outcome measures	Not included in the search strategy
Filter	English AND Published from 2009 to 2013 AND (animal experiment OR animal tissue OR animal model)

**Table 3:** Basic characteristics of the animal models used in the included studies.

Study	Sex/model species	Induction of AD-like skin	Barrier disruption	Investigation site
Qi et al. [27]	F/hairless mouse	DNCB	N/A	Dorsal skin
Lee et al. [14]	M/NC/Nga mouse	DfE	SDS	Dorsal skin, ears
Choi et al. [15]	F/NC/Nga mouse	DfE	N/A	Dorsal skin
Sohn et al. [28]	M/BALB/c mouse	DNCB	N/A	Dorsal skin
Wu et al. [29]	M/NC/Nga mouse	DNFB	N/A	Dorsal skin
Yang et al. [16]	F/NC/Nga mouse	DNCB	N/A	Dorsal skin, ears
Sung et al. [30]	M/NC/Nga mouse	DfE	SDS	Dorsal skin, ears
Nam et al. [31]	M/C57BL/6 mouse	DNFB	N/A	Abdominal skin, ears
Choi et al. [24]	F/BALB/c mouse	DNCB, DfE, and skin injury	N/A	Ears
Sung et al. [9]	M/NC/Nga mouse	DfE	SDS	Dorsal skin, ears
Lee et al. [22]	M/NC/Nga mouse	DfE	SDS	Dorsal skin, ears
Ngatu et al. [17]	M/NC/Nga mouse	TNCB	N/A	Abdominal skin, ears
Yang et al. [18]	F/NC/Nga mouse	DNCB	SDS	Dorsal skin, ears
Hwang et al. [25]	F/BALB/c mouse	DNCB, DfE, and skin injury	N/A	Ears
Choi et al. [32]	M/NC/Nga mouse	DNCB	N/A	Dorsal skin, ears
Sung et al. [33]	M/NC/Nga mouse	DNCB and DfE	SDS	Dorsal skin, ears
Kang and Shin [34]	M/NC/Nga mouse	DNCB	N/A	Dorsal skin
Sung et al. [35]	M/NC/Nga mouse	DfE	SDS	Dorsal skin, ears
Park et al. [10]	F/NC/Nga mouse	DNCB	N/A	Dorsal skin, right ear
Yang et al. [36]	M/NC/Nga mouse	DfE	N/A	Dorsal skin, ears
Kim et al. [37]	F/BALB/c mouse	DNCB	SDS	Dorsal skin
Choi et al. [38]	M/NC/Nga mouse	DNCB	N/A	Dorsal skin, ears

DNCB: 1-chloro-2,4-dinitrobenzene, DfE: D. farinae extract, DNFB: 2,4-dinitrofluorobenzene, TNCB: 2,4,6-trinitrochlorobenzene, N/A: not applicable, SDS: sodium dodecyl sulfate, M: male, and F: female.

**Table 4:** Investigated herbs, results, and suggested mechanisms of action in the included studies.

Ref. number	Herb	Outcomes and results	Suggested mechanisms
[14]	*Bambusae caulis *	TEWL↓ serum IgE↓ eosinophil↓ spleen IFN-*γ*↑ TNF-*α*↓ IL-4↓ IL-13↓	Suppression of Th2 response and promotion of Th1 response

[15]	*Broussonetia kazinoki *	Plasma IgE↓ IL-4↓ skin mast cell↓	Suppression of Th2 response

[28]	*Alnus japonica *	Clinical score↓ serum IgE↓ eosinophil↓ skin IL-4↓ IL-5↓ IL-13↓ iNOS↓ COX-2↓	Suppression of Th2 response

[29]	Korean red ginseng	Scratching↓ serum IgE↓ IL-4↓ IL-10↓	Suppression of Th2 response

[16]	*Cordyceps bassiana *	Clinical score↓ epidermal thickness↓ serum IgE↓ histamine↓ skin IFN-*γ*↓ IL-4↓ mast cell↓	Suppression of both Th1 and Th2 responses

[30]	*Chelidonium majus *	Clinical score↓ ear thickness↓ scratching↓ serum IgE↓ IL-4↓ TNF-*α*↓	Suppression of Th2 response

[31]	*Cinnamomum cassia *	Serum IgE↓ histamine↓ TNF-*α*↓ skin IL-4↓ TNF-*α*↓ TARC↓	Suppression of Th2 response

[24]	*Terminalia chebula *Retzius	Ear thickness↓ skin inflammatory cells↓ eosinophils↓ ear IL-31↓ T-bet positive cell↑ MMP-9↓	Suppression of Th2 response and promotion of Th1 response

[9]	*Lindera obtusiloba *	Ear thickness↓ serum IgE↓ DfE specific IgE↓ histamine↓ ear mast cell↓ IL-4↓ IL-13↓ IL-31↓ TNF-*α*↓	Suppression of Th2 response

[22]	*Rehmannia glutinosa *	Clinical score↓ ear thickness↓ serum IgE↑ histamine↓ ear IL-4↓ TNF-*α*↓ TARC↓ MDC↓ RANTES↓ ICAM-1↓ VCAM-1↓	Suppression of Th2 response

[17]	*Angelicae Dahuricae Radix *	Clinical score↓ plasma IgE↓ histamine↓	Suppression of Th2 response

[18]	*Vernonia amygdalina *	Clinical score↓ ear thickness↓ scratching serum IgE↓ IL-4↓ IL-5↓ MCP-1↓ eotaxin↓	Suppression of Th2 response

[25]	*Chrysanthemum boreale *Makino	Clinical score↓ ear thickness↓ scratching↓ serum IgE↓ TNF-*α*↓ IL-4↓	Suppression of Th2 response

[32]	Mycelium of* Phellinus linteus *	Clinical score↓ ear thickness↓ serum IgE↓ DfE specific IgE↓ total IgG— ear IL-12↓ IFN-*γ*↓ IL-4↓ IL-5↓ IL-10↓ IL-13↓ TNF-*α*↓ CCL4↓ CCL22↓ CCL17— CCL20— eotaxin— IL-2↓	Suppression of both Th1 and Th2 responses

[33]	*Psidium guajava *	Clinical score↓ serum IgE↓ TARC↓ IL-10↑ ear IFN-*γ*↓ TNF-*α*↓ IL-4↓ IL-5↓ IL-13↓	Suppression of both Th1 and Th2 responses and upregulation of IL-10

[34]	*Drynaria fortunei *	Clinical score↓ ear thickness↓ serum IgE↓ IgG1↓ IgG2a— IL-4↓ IL-6↓ TNF-*α*↓ ear IFN-*γ*— IL-4↓ TNF-*α*↓ IL-6↓ ICAM-1— VCAM-1	Suppression of Th2 response

[35]	*Schisandra chinensis *	Clinical score↓ scratching↓ serum IgE↓ IgM↓ histamine↓ skin histamine receptors↓ spleen IL-4↓ IL-5↓ Fc*ε*RI*β*↓	Suppression of Th2 response

[10]	*Illicium verum *	Clinical score↓ ear thickness↓ serum IgE↓ histamine↓ IL-6↓ ICAM-1↓ ear IFN-*γ*↑ IL-4↓ IL-6↓ TNF-*α*↓ TARC↓ RANTES↓ ICAM-1↓ VCAM-1↓	Suppression of Th2 response and promotion of Th1 response

[36]	*Chrysanthemum indicum *L.	Clinical score↓ ear thickness↓ serum IgE↓ IgG1↓ IFN-*γ*↓ IL-4↓ skin eosinophil↓ mast cell↓ IFN-*γ*↓ IL-4↓ IL-13↓ IL-4 : IFN-*γ* ratio↓	Suppression of both Th1 and Th2 responses and balancing of Th1/Th2 cell responses

[37]	*Catalpa ovata *	Clinical score↓ serum IgE↓ skin mast cell↓ IL-4↓ IL-5↓ IL-6↓ IL-13↓ IL-1b↓ TNF-*α*↓	Suppression of Th2 response

[38]	*Astragalus membranaceus *	Epidermal thickness↓ dermal thickness↓ serum IgE↓ IL-4↓ IL-5↓ IL-6↓ IL-13↓ TNF-*α*↓ skin NF-*κ*B↓	Suppression of Th2 response

[11]	*Pleurotus eryngii *	Clinical score↓ ear thickness↓ serum IgE↓ TARC↓ skin mast cell↓ ear IFN-*γ*↓ TNF-*α*↓ IL-4↓ IL-5↓ IL-13↓	Suppression of both Th1 and Th2 responses

“—”: no change, TEWL: transepidermal water loss, Ig: immunoglobulin, IFN: interferon, IL: interleukin, TNF: tumor necrosis factor, iNOS: inducible nitric oxide synthase, COX: cyclooxygenase, TARC: thymus and activation-regulated chemokine, MMP: matrix metalloproteinase, MDC: macrophage-derived chemokine, RANTES: regulated on activation normal T-cell expressed and secreted, ICAM: intercellular adhesion molecule, VCAM: vascular adhesion molecule, MCP: monocyte chemoattractant protein, CCLCC: chemokine ligand, Fc*ε*RI: high-affinity IgE receptor, and NF-*κ*B: nuclear factor-*κ*B.
